# Dual-trigger improves the outcomes of in vitro fertilization cycles in older patients with diminished ovarian reserve: A retrospective cohort study

**DOI:** 10.1371/journal.pone.0235707

**Published:** 2020-07-06

**Authors:** Chyi-Uei Chern, Ju-Yueh Li, Kuan-Hao Tsui, Peng-Hui Wang, Zhi-Hong Wen, Li-Te Lin

**Affiliations:** 1 Department of Obstetrics and Gynecology, Kaohsiung Veterans General Hospital, Kaohsiung City, Taiwan; 2 Institute of BioPharmaceutical sciences, National Sun Yat-sen University, Kaohsiung City, Taiwan; 3 Department of Obstetrics and Gynecology, National Yang-Ming University School of Medicine, Taipei, Taiwan; 4 Department of Pharmacy and Master Program, College of Pharmacy and Health Care, Tajen University, Pingtung County, Taiwan; 5 Department of Obstetrics and Gynecology, Taipei Veterans General Hospital, Taipei, Taiwan; 6 Department of Medical Research, China Medical University Hospital, Taichung City, Taiwan; 7 Department of Marine Biotechnology and Resources, National Sun Yat-sen University, Kaohsiung City, Taiwan; Universita degli Studi dell’Insubria, ITALY

## Abstract

**Background:**

Dual-trigger for final oocyte maturation has been applied on the women with poor ovarian response or diminished ovarian reserve. However, the results were controversial. The Patient-Oriented Strategies Encompassing IndividualizeD Oocyte Number (POSEIDON) stratification is a set of newly established criteria for low prognosis patients. The aim of this study was to examine the effectiveness of dual-trigger for final oocyte maturation on the in vitro fertilization (IVF) outcomes of patients who fulfill the POSEIDON group 4 criteria.

**Methods:**

This retrospective cohort study investigated 384 cycles fulfilling the POSEIDON group 4 criteria. The patients underwent IVF treatment using the gonadotropin-releasing hormone (GnRH) antagonist protocol. The study group contained 194 cycles that received dual-trigger (human chorionic gonadotropin [hCG] plus GnRH-agonist) for final oocyte maturation. The control group included 114 cycles where final oocyte maturation was performed with only hCG. Baseline characteristics and cycle parameters, as well as IVF outcomes of both groups were compared.

**Results:**

Baseline characteristics were similar between the dual trigger group and the control group. In terms of IVF outcomes, the dual trigger group demonstrated significantly higher number of retrieved oocytes, metaphase II oocytes, fertilized oocytes, day-3 embryos, and top-quality day-3 embryos. A statistically significant improvement in clinical pregnancy rate and live birth rate was also observed in the dual trigger group.

**Conclusions:**

Our data suggests that dual trigger for final oocyte maturation might improve clinical pregnancy rates and live birth rates of IVF cycles in patients fulfilling the POSEIDON group 4 criteria.

## Introduction

The process of in vitro fertilization (IVF) involves hyperstimulation of ovaries with gonadotropins to mimic the natural cycle in producing mature oocytes. In the normal menstrual cycle, oocyte maturation occurs following the luteinizing hormone (LH) and smaller follicle stimulating hormone (FSH) surge that happens during mid-cycle. Traditionally, human chorionic gonadotropin (hCG) has been the trigger of choice for oocyte maturation due to its molecular and biological similarity with LH [1]. However, its half-life is much longer than LH, lasting for approximately days [2]. This contributes to the occurrence of ovarian hyperstimulation syndrome (OHSS) in high responders. Also, hCG lacks FSH activity, which plays a role in the in vitro maturation of oocytes [3].

Gonadotropin-releasing hormone (GnRH) agonists were first suggested for final oocyte maturation by Gonen et al. in 1990, as it is able to trigger endogenous release of both FSH and LH [4]. With a shorter mean duration of LH surge of about 34 hours, it is similar to the natural cycle duration of 48 hours [5], effectively reducing the incidence of OHSS in high responders [6, 7]. However, some problems surfaced with the substitution of GnRH-agonists as trigger. Kummer et al. discovered that the risk of empty follicle syndrome was increased following isolated GnRH-agonist trigger due to a suboptimal LH surge [8]. Additionally, increased early pregnancy loss and decreased rates of ongoing pregnancy were noted by multiple studies [9, 10]. As such, the idea of a dual trigger was developed [11]. Indeed, the hCG component of dual trigger could serve as a rescue trigger in case of poor response to GnRH-agonist, which occurs in about 2.71% of a study population [12].

Since its development, multiple investigations have shown the benefits of using a dual trigger for final oocyte maturation in normal responders [11, 13], including an improvement in total number of retrieved oocytes, MII oocytes, rates of embryo implantation, clinical pregnancy, and live birth rates [14]. Evidence from available meta-analysis in 2018 involving four studies including 527 patients found a significantly improved clinical pregnancy rate following dual trigger [15]. However, for poor ovarian responders (PORs), the situation is less clear cut.

Women with poor ovarian response are those who have a reduced number of follicles responsive to FSH, resulting in poor IVF outcomes [16]–posing a grave challenge to clinicians worldwide. Numerous criteria have been proposed for the definition of POR [17], but none was established as the international standard to define POR until the creation of the Bologna criteria in 2011 [18]. Presence of much heterogeneity lead to strong criticism about the clinical application of the Bologna criteria [19], triggering the development of the POSEIDON (Patient-Oriented Strategies Encompassing IndividualizeD Oocyte Number) criteria in 2016 [20]. The POSEIDON criteria brings forward the concept of “low prognosis”, and classifies all patients according to 3 factors: oocyte quality as demonstrated by age, oocyte quantity as represented by ovarian biomarkers of anti-Müllerian hormone (AMH) and/or antral follicle count (AFC), and finally ovarian response, dependent on the number of oocytes retrieved given a previous ovarian stimulation cycle. As such, it is able to aid the clinician in formulating a more tailored treatment plan for the individual groups. The focus of our study is POSEIDON group 4, with patients who are ≥ 35 years old, with AFC < 5 and/or AMH < 1.2 ng/mL. However, it is of note that the patients in POSEIDON group 4 do not display elevated FSH, which is a distinguishing feature of premature ovarian insufficiency.

Based on the existing evidence of improved reproductive outcomes in normal responders, and the theoretical advantage of a more physiologic trigger while reducing risk of poor response to GnRH-agonist alone, we hypothesized that similar benefits can be obtained using dual-trigger in a low prognosis group like POSEIDON 4. Therefore, we attempted to investigate the feasibility of utilizing dual-trigger for final oocyte maturation in improving IVF outcomes of this population.

## Materials and methods

### Study design and participants

The retrospective cohort study was performed at the reproductive medical center of Kaohsiung Veterans General Hospital, in Kaohsiung, Taiwan from January 2012 through December 2017. The study protocol was approved by the institutional review board at Kaohsiung Veterans General Hospital, with the identifier VGHKS19-CT8-05, and conforms to the “Declaration of Helsinki for Medical Research involving Human Subjects.” The need for consent was waived by the ethics committee due to its retrospective design. Data was compiled from electronic medical records and cycle flow sheets for patients within the study period. Patients who underwent IVF cycles and fulfilled the criteria for POSEIDON group 4 (age ≥ 35 years, with AFC < 5 and/or AMH < 1.2 ng/mL) were included in this study. IVF is the treatment of choice for this group of patients characterized by advanced maternal age and low ovarian reserve due to its relatively higher success rate compared to other treatment modalities. A total of 384 cycles met the criteria during the study period. Exclusion criteria were as follows: (i) Patients who did not receive GnRH antagonist protocol, (ii) patients with premature ovarian insufficiency, (iii) patients over the age of 45, (iv) cancer patients who have received chemotherapy or radiotherapy, and (v) patients with incomplete data. Following application of the exclusion criteria, a total of 308 cycles were included for study and then divided into dual-trigger and hCG trigger groups. The choice of hCG alone or dual-trigger depended on the physician, with cycles before 2015 being mostly hCG-only trigger and those after 2015 being mostly dual-trigger. In the dual-trigger group (n = 194), patients received final oocyte maturation with GnRH-agonist and hCG. In the hCG trigger group (n = 114), patients received only hCG for final oocyte maturation.

### Treatment protocol

Only patients receiving GnRH antagonist protocol were included in this study. Following a baseline hormone screen and transvaginal ultrasound for antral follicles, controlled ovarian stimulation was initiated within 5 days of the menstrual cycle, with recombinant follicle stimulating hormone (rFSH, Gonal-F, Merck Serono S.p.A., Modugno, Italy), rFSH plus recombinant luteinizing hormone (Pergoveris, Merck Serono SA, Aubonne, Switzerland), human menopausal gonadotropin (Merional, IBSA Institut Biochimique S.A., Lamone, Switzerland), or corifollitropin alfa (Elonva, Vetter Pharma-Fertigung GmbH & Co, KG, Ravensburg, Germany). Furthermore, addition of growth hormone (Saizen, Merck Serono SA, Aubonne, Switzerland) was given during controlled ovarian stimulation at physician’s discretion.

Patient response was monitored during the IVF cycle with serial transvaginal ultrasound for follicular measurements and hormone profiles. Dosage was adjusted depending on follicular response and previous response to gonadotropins. Daily GnRH antagonist injections (Cetrotide 0.25mg, Pierre Fabre Medicament Production, Aquitaine Pharm International, Idron, France or Orgalutran 0.25mg, Vetter Pharma-Fertigung GmbH & Co, KG, Ravensburg, Germany) were administered when the leading follicle reaches 12–14 mm in diameter, up till the date of final oocyte maturation. Patient then either received dual-trigger with combined recombinant hCG (Ovidrel 250μg, Merck Serono S.p.A., Modugno, Italy) with GnRH agonist (Lupro 2mg, Nang Kuang Pharmaceutical Co, Ltd., Tainan, Taiwan) or recombinant hCG alone. Trigger is given when at least one leading follicle reaches a mean diameter of 18 mm.

Transvaginal ultrasound-guided oocyte retrieval was performed 36 h following administration of trigger. Whether fertilization was conducted by conventional IVF or intracytoplasmic sperm injection (ICSI) depended on semen analysis results or prior fertilization condition. Embryos were evaluated and graded according to the criteria established by the Istanbul consensus workshop [21]. The number of blastomeres, the percentage of fragmentation and the variation in blastomere symmetry were assessed. Top-quality cleavage stage embryos were determined as those with the following features: six cells or greater on day 3, less than 10% fragmentation and symmetric blastomeres. All embryos were cryopreserved by vitrification under a freeze-all strategy on the third day after oocyte retrieval for subsequent frozen embryo transfer. As such, no embryo qualified for preimplantation genetic testing for aneuploidies (PGT-A). Vitrification was carried out via a two-step exposure to equilibrium and vitrification solutions. The embryo is first exposed to the equilibrium solution for 15 minutes, followed by exposure to the vitrification solution for 1 minute and 30 seconds. The embryo is then loaded onto the propylene strip of Cryotop (Kitazato, Bio Pharma Co., Tokyo, Japan) in an open system with minimal solution, and then rapidly plunged into liquid nitrogen at -196°C. An artificial cycle was used for endometrial preparation. Endometrium was prepared with daily oral estradiol (Ediol 8mg, Synmosa Biopharma Corporation, Hsinchu County, Taiwan) and estradiol gel (Oestrogel gel, Besins, Drogenbos, Belgium) beginning before cycle day 5.

When the endometrial thickness reaches at least 8mm, daily progesterone, including intravaginal gel (Crinone 8% gel, Merck Serono, Hertfordshire, UK) and oral dydrogesterone (Duphaston 40mg, Abbott, Olst, the Netherlands), were given simultaneously as luteal phase support. Additionally, aspirin (ASPIRIN PROTECT 100mg, Bayer AG, Leverkusen, Germany) was also prescribed routinely. Following assisted-hatching via laser zona drilling, the frozen-thawed embryos were transferred under transabdominal sonographic assistance. Progesterone supplementation was administered until 8–10 gestational weeks upon confirmation of pregnancy.

### Outcome measures

The primary outcome measure was live birth rate, defined as the delivery of a viable fetus beyond 24 weeks of gestation. Secondary outcome measures included the number of retrieved oocytes, number of mature oocytes, number of fertilized oocytes, number of day-3 embryos, number of top-quality day-3 embryos, implantation rate (calculated from the number of gestation sacs with fetal heart seen on ultrasound scan divided by the total number of transferred embryos), cancellation rate, miscarriage rate, and clinical pregnancy rate. Cancellation rate was defined as those with no retrieved oocytes, or no viable embryos, while miscarriage rate refers to pregnancy loss before 24 weeks of gestation, and clinical pregnancy rate was defined by the presence of a fetal heartbeat at 6–7 weeks of a pregnancy.

### Statistical analysis

Kolmogovor-Smirnov test was used to evaluate normality of quantitative variables. Quantitative variables were evaluated using the independent t-test. Chi-Square test was used to evaluate categorical variables. Odds ratios (ORs) and 95% confidence intervals (CIs) of live birth and clinical pregnancy were assessed using generalized estimating equations (GEEs), after adjusting for confounders. We adopted GEEs to account for correlations between multiple cycles from the same patient. Key factors including age, BMI, infertility duration, previous IVF attempts, basal FSH, AFC, AMH, number of pre-ovulatory follicles > 10 mm on trigger day, and number of pre-ovulatory follicles > 14 mm on trigger day, were identified as confounders for analyses. Analyses were performed using the Statistical Package for Social Sciences (SPSS) version 20.0 (Chicago, IL, USA). *P* < 0.05 was considered statistically significant.

## Results

**Fig 1:**
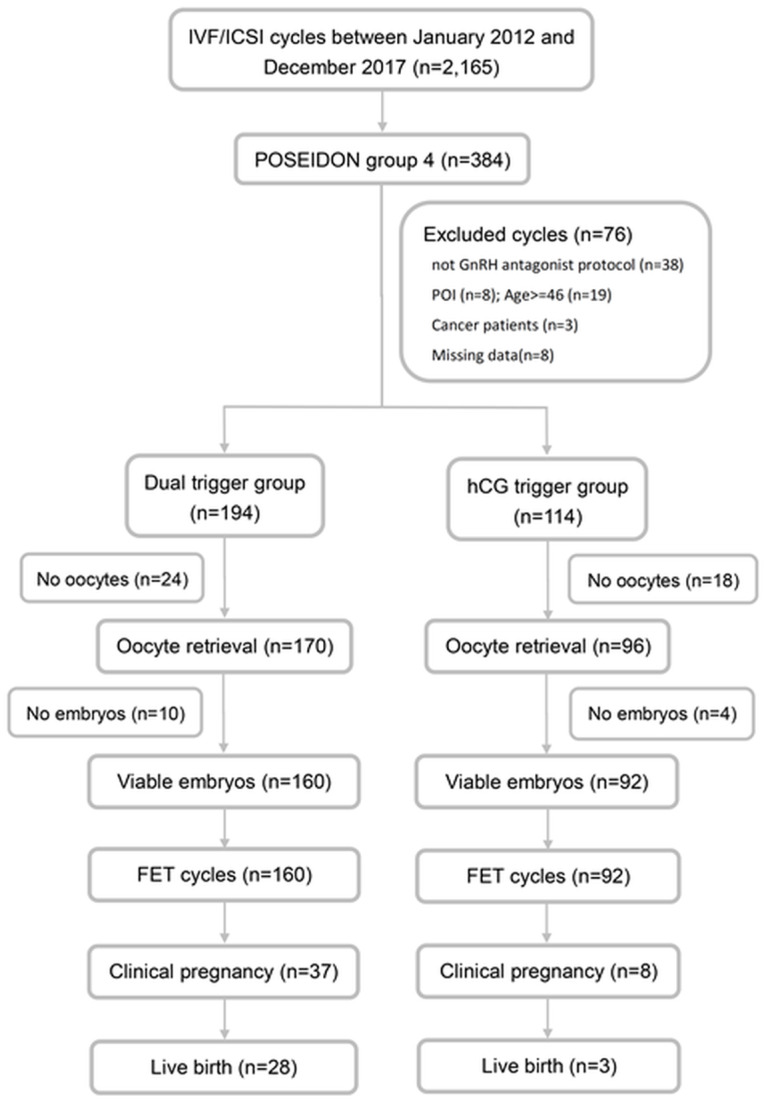
Flow chart of the study design. IVF, *in vitro* fertilization; ICSI, intracytoplasmic sperm injection; GnRH, gonadotropin-releasing hormone; POI, primary ovarian insufficiency; FET, frozen embryo transfer.

As shown in Fig 1, out of 2,165 IVF cycles, 384 cycles fulfilled the POSEIDON group 4 criteria. Of the 384 cycles, 38 were not treated using a GnRH antagonist protocol, 8 were diagnosed with premature ovarian insufficiency, 19 with advanced maternal age of more than 45 years old, 3 were cancer patients who underwent chemotherapy and/or radiotherapy, and 8 had incomplete dataset. Those cycles were excluded from the study. Of the remaining 194 cycles in the dual-trigger group and 114 in the hCG-only trigger group, a further 34 cycles were excluded in the dual-trigger group, and 22 cycles excluded in the hCG-only group due to no retrieved oocytes or no viable embryos. As such, 160 frozen-thawed embryo transfer cycles from the dual-trigger group and 92 frozen-thawed embryo transfer cycles from the hCG-only group were available for analysis.

Comparisons between the populations of two groups revealed no difference in patient age, body mass index, infertility duration, previous IVF attempts, primary or secondary infertility, cause of infertility, basal FSH, basal luteinizing hormone (LH), basal estradiol (E2), AFC and AMH (Table 1).

**Table 1:** Baseline characteristics of older patients with diminished ovarian reserve (POSEIDON group 4) with dual-trigger or hCG trigger.

Parameters	Dual trigger (n = 194)	hCG trigger (n = 114)	*p* value
Age (years)	40.0±2.7	40.0±2.8	0.948
Body mass index (kg/m^2^)	22.9±3.5	22.4±2.9	0.170
Infertility duration (years)	5.6±4.6	5.1±4.3	0.402
Previous IVF attempts (n)			0.340
0	31.4% (61/194)	39.5% (45/114)	
1~2	32.5% (63/194)	27.2% (31/114)	
≧3	36.1% (70/194)	33.3% (38/114)	
Types of infertility (%)			0.335
Primary infertility	55.7 (108/194)	50 (57/114)	
Secondary infertility	44.3 (86/194)	50 (57/114)	
Cause of infertility			0.713
Tubal factor	11.3% (22/194)	8.8% (10/114)	
Male factor	20.6% (40/194)	23.7% (27/114)	
Endometriosis	14.4% (28/194)	17.5% (20/114)	
Uterine factor	10.8% (21/194)	11.4% (13/114)	
Multiple factors	30.9% (60/194)	31.6% (36/114)	
Unexplained	11.9% (23/194)	7.0% (8/114)	
Basal FSH (IU/l)	6.3±3.8	7.0±3.3	0.158
Basal LH (IU/l)	3.2±1.2	3.2±1.1	0.957
Basal E2 (pg/mL)	29.5±10.8	31.7±12.3	0.128
Antral follicle counts (n)	2.4±1.3	2.5±1.7	0.425
Anti-Müllerian hormone (ng/mL)	0.48±0.26	0.47±0.31	0.857

Data are presented as mean ± standard deviation and %.

IVF, *in vitro* fertilization; FSH, follicle-stimulating hormone; LH, luteinizing hormone; E2, estradiol

Cycle characteristics between the two groups are presented in Table 2. There were no differences in terms of stimulation duration, types of gonadotropins, total gonadotropin dose and percentage of growth hormone supplementation. Pre-ovulatory follicles > 10 mm (3.7±2.6 vs. 3.4±1.5, *p* = 0.573) and pre-ovulatory follicles > 14 mm (2.9±2.2 vs. 2.7±0.9, *p* = 0.191) on trigger day were similar between the two groups. However, the number of retrieved oocytes (3.3±2.7 vs. 1.6±1.5, *p*<0.001), metaphase II oocytes (2.6±2.0 vs. 1.3±1.0, *p*<0.001), fertilized oocytes (2.4±2.1 vs. 1.2±1.0, *p*<0.001), day-3 embryos (2.2±1.9 vs. 1.2±1.0, *p*< 0.001) and top-quality day-3 embryos (0.9±1.3 vs. 0.2±0.5, *p*<0.001) were significantly higher in the dual-trigger group compared with the hCG-only group. Moreover, methods of fertilization, fertilization rate and cancellation rate were similar between the two groups.

**Table 2:** Cycle characteristics of older patients with diminished ovarian reserve (POSEIDON group 4) with dual-trigger or hCG trigger.

Parameters	Dual trigger (n = 194)	hCG trigger (n = 114)	*p* value
Stimulation duration (days)	10.6±2.2	10.4±2.7	0.445
Types of gonadotropins			0.744
rFSH	5.7% (11/194)	7.9% (9/194)	
rFSH + rLH	56.2% (109/194)	54.4% (62/194)	
HMG	38.1% (74/194)	37.7% (43/194)	
Gonadotropin dosage (IU)			
with corifollitropin alfa	1751.4±708.9	1561.4±676.6	0.316
without corifollitropin alfa	2931.5±779.9	2729.7±1109.0	0.129
GH supplementation (%)	37.1% (72/194)	40.4% (46/114)	0.573
Pre-ovulatory follicles > 10 mm on trigger day	3.7±2.6	3.4±1.5	0.191
Pre-ovulatory follicles > 14 mm on trigger day	2.9±2.2	2.7±0.9	0.220
No. of oocytes retrieved (n)	3.3±2.7	1.6±1.5	<0.001
No. of metaphase II oocytes (n)	2.6±2.0	1.3±1.0	<0.001
No. of fertilized oocytes (n)	2.4±2.1	1.2±1.0	<0.001
Methods of fertilization			0.810
IVF	62.9% (107/170)	61.5% (59/96)	
ICSI	37.1% (63/170)	38.5% (37/96)	
Fertilization rate (%)	74.3±37.4	77.8±39.4	0.440
No. of day 3 embryos (n)	2.2±1.9	1.2±1.0	<0.001
No. of top-quality day 3 embryos (n)	0.9±1.3	0.2±0.5	<0.001
Cancellation rate (%)	17.5% (34/194)	19.3% (22/114)	0.697

Data are presented as mean ± standard deviation and %.

rFSH, recombinant follicle-stimulating hormone; rLH, recombinant luteinizing hormone; HMG, human menopausal gonadotropin; GH, growth hormone; IVF, *in vitro* fertilization; ICSI, intracytoplasmic sperm injection

Pregnancy outcomes between the two groups are presented in Table 3. Endometrium thickness was similar between the two groups. However, the number of transferred embryos (2.1±1.0 vs. 1.4±0.8, *p*<0.001) and percentage of at least one top-quality embryos transfer (62.5% vs. 23.9%, *p*<0.001) were significantly higher in the dual-trigger group than in the hCG-only group. Of note, the dual-trigger group performed superiorly in terms of implantation rate (14.4±30.0% vs. 5.4±18.8%, *p* = 0.004), clinical pregnancy rate (23.1% vs. 8.7%, *p* = 0.004) and live birth rate (17.5% vs. 5.4%, *p* = 0.006). Furthermore, there was no significant difference in miscarriage rate between the two groups.

**Table 3:** Pregnancy outcomes of older patients with diminished ovarian reserve (POSEIDON group 4) with dual-trigger or hCG trigger.

Parameters	Dual trigger (n = 160)	hCG trigger (n = 92)	*p* value
Endometrium thickness	10.4±1.2	10.6±1.3	0.337
No. of transferred embryos	2.1±1.0	1.4±0.8	<0.001
At least one top-quality embryo transfer (%)	62.5% (100/160)	23.9% (22/92)	<0.001
Implantation rate (%)	14.4±30.0	5.4±18.8	0.004
Clinical pregnancy rate (%)	23.1% (37/160)	8.7% (8/92)	0.004
Live birth rate (%)	17.5% (28/160)	5.4% (5/92)	0.006
Miscarriage rate (%)	24.3% (9/37)	37.5% (3/8)	0.445

Data are presented as mean ± standard deviation and %.

GEEs were performed to determine whether dual-trigger use had a beneficial effect on clinical pregnancy rate and live birth rate in Table 4. Confounding variables such as age, BMI, duration of infertility, previous IVF attempts, basal FSH, AFC, AMH, number of pre-ovulatory follicles > 10 mm on trigger day, and number of pre-ovulatory follicles > 14 mm on trigger day were included in the analysis. The analysis showed that dual-trigger use was positively associated with live birth rate (OR = 3.16, 95% CI 1.06–9.38, *p* = 0.039) and clinical pregnancy rate (OR = 4.30, 95% CI 1.38–13.43, *p* = 0.012). Furthermore, AMH was shown to be a positive independent factor affecting clinical pregnancy rate (OR = 7.20, 95% CI 1.31–39.61, *p* = 0.023).

**Table 4:** Analyses of factors affecting live birth and clinical pregnancy in older patients with diminished ovarian reserve (POSEIDON group 4) using generalized estimating equations.

	Live birth		Clinical pregnancy	
	Adjusted OR (95% CI)	*p* value	Adjusted OR (95% CI)	*p* value
Dual vs. hCG trigger	3.16(1.06–9.38)	0.039	4.30(1.38–13.43)	0.012
Age (years)	0.93(0.81–1.08)	0.353	0.95(0.83–1.10)	0.502
BMI (kg/m^2^)	1.02(0.88–1.19)	0.758	1.02(0.90–1.16)	0.790
Infertility duration (years)	0.98(0.89–1.08)	0.665	0.95(0.87–1.04)	0.286
Previous IVF attempts (n)	0.91(0.46–1.80)	0.792	1.09(0.63–1.88)	0.756
Basal FSH (IU/l)	0.89(0.77–1.04)	0.149	0.93(0.82–1.06)	0.265
AFC (n)	1.10(0.82–1.48)	0.535	1.01(0.73–1.40)	0.951
AMH (ng/mL)	2.37(0.48–11.64)	0.287	7.20(1.31–39.61)	0.023
No. of pre-ovulatory follicles > 10 mm on trigger day (n)	1.36(0.57–3.23)	0.484	1.51(0.95–2.40)	0.082
No. of pre-ovulatory follicles > 14 mm on trigger day (n)	0.74(0.30–1.80)	0.501	0.59(0.34–1.06)	0.066

OR, odds ratio; CI, confidence interval; BMI, body mass index; IVF, *in vitro* fertilization; FSH, follicle- stimulating hormone; AFC, antral follicle count; AMH, anti-Müllerian hormone.

## Discussion

This retrospective cohort study is the first to assess the effects of dual-trigger on IVF outcomes in patients fulfilling the POSEIDON group 4 criteria. Our study showed that dual-trigger is superior to hCG administration alone for final oocyte maturation in producing increased numbers of retrieved oocytes, metaphase II oocytes, fertilized oocytes, day-3 embryos and top-quality day-3 embryos. Clinical pregnancy rate and live birth rate were also improved with dual-trigger administration. Moreover, the analysis using GEEs revealed a 4.30-fold increase in clinical pregnancy (95% CI 1.38–13.43, *p* = 0.012) and a 3.16-fold increase in live birth (95% CI 1.06–9.38, *p* = 0.039) in the POSEIDON group 4 patients with dual trigger compared to those using hCG trigger alone. Similar outcomes have been obtained in the study of patients with diminished ovarian reserve or poor ovarian reserve in recent years, showing a beneficial effect of dual-trigger in improving IVF outcomes. Lin et al. confirmed in a recent retrospective cohort study involving 427 GnRH antagonist IVF cycles with fresh embryo transfer that dual-triggering significantly increases the live birth rate (26.9% vs 14.5%, *p* = 0.014), clinical pregnancy rate (33.0% vs. 20.7%, *p* = 0.035), and fertilization rate (73.1% vs. 58.6%, *p* = 0.015) in women with diminished ovarian reserve, compared to hCG-alone trigger [22]. In an even larger retrospective cohort study with 1389 IVF cycles fulfilling the Bologna criteria, utilizing the progesterone-primed ovarian stimulation protocol, Zhang et al. also reports significantly higher number of oocytes collected (*p* < 0.001) with improved number of mature oocytes (*p* < 0.001) [23]. However, other studies have found dual-trigger to be ineffective at improving IVF outcomes for this population. Eser et al., in a case control study involving 47 dual-trigger and 62 hCG-only trigger cases fulfilling the Bologna criteria for POR, discovered no statistical difference between the two groups with reference to implantation rate, biochemical pregnancy rate, clinical pregnancy rate, and ongoing pregnancy rate [24]. Due to the divergence of opinion on the effectiveness and clinical utility of dual-trigger for final oocyte maturation, large-scale randomized controlled trials are required to reach a verdict.

In combining GnRH-agonist and hCG for the final oocyte trigger, we are in essence enjoying the best of both worlds. Despite being molecularly similar, hCG and LH elicit different gene expressions. LH tends towards cellular growth, which supports embryo development and survival, whereas hCG enhances apoptosis [25, 26]. hCG administration alone also does not produce FSH activity, while GnRH-agonist releases an endogenous FSH and LH surge, resulting in a more physiologic response.

In vitro studies have highlighted the role of FSH in oocyte maturation [3], via the increased production of epiregulin (Ereg) and amphiregulin (Areg) [27], members of the epidermal growth factor-like (EGF) family, which have been shown to mediate the LH signal and participate in cumulus expansion and oocyte maturation [28]. This has been confirmed in vitro experiments, where Ereg and Areg presence in maturation medium helps to improve the maturation rate of human GV oocytes [29]. Animal studies have also shown that FSH has the independent ability of inducing ovulation [30], perhaps by stimulating plasminogen activator activity, which converts plasminogen into active protease plasmin, helping to weaken the follicular wall and aiding rupture and oocyte dissociation [3, 31, 32]. Also, the FSH surge induces LH formation on luteinized granulosa cells, promoting oocyte maturation and cumulus expansion [33].

Additionally, direct GnRH receptor activation as identified by Raga et al. could also have an effect on preimplantation embryonic development that is unrelated to FSH activity [34]. The GnRH receptor expression was found to be greatest in granulosa cells [35], and in rat models, administration of GnRH-agonists induced an increase in receptor levels in a dose-dependent manner, whereas LH decreased GnRH receptor mRNA levels [36]. Administration of GnRH-agonist trigger has also demonstrated the retrieval of more MII oocytes (16%) [9]. As such, the triggering cocktail of hCG, FSH, LH and GnRH-agonist serves to provide the benefits of a multi-faceted approach.

Subgroups include those with low proportion of mature oocytes (< 66%) per number of oocytes retrieved [37], where it was demonstrated that patients who received dual-trigger had significantly higher number of MII oocytes (6.5 vs. 3.6, *p*< 0.008), number of oocytes retrieved (69.7% vs. 47.1%, *p* < 0.03), and a higher number of top-quality embryos (3.1 vs. 1, *p* < 0.02) [38]. Moreover, those with history of poor fertilization, as defined by fertilization rate < 20% in at least two prior ICSI cycle, are also a potential benefit of such a combination. In their retrospective cohort study, Elias et al. found in 2017 that the mean fertilization rate in the combined trigger group was found to be significantly higher 16.4% (95% CI 7.58%–25.2%), with higher oocyte maturity (82.1% vs. 69.8%), higher clinical pregnancy (27.5% vs. 5.67%), and higher live birth rates (20.2% vs. 3.46%) compared to the hCG trigger group [39].

The relatively small sample size of this study along with its retrospective design poses major limitations. The retrospective nature of this study makes it more susceptible to selection bias. Selection of cases for dual-trigger or hCG only trigger and growth hormone use was up to the physician’s discretion, predisposing it to possibility of bias. Also, as most of the cases utilized dual-trigger following 2015, while those prior to 2015 utilized hCG-only trigger, we cannot rule out the effects of chronological bias. Another shortcoming of our study is that although all cycles were antagonist cycles with similar types of gonadotropins between both groups, using various types of gonadotropins still leads to possibility of bias. The strength of our study is that all clinical decisions and oocyte pick-ups were performed by the same physician, leading to less variability in performance.

## Conclusions

Our data suggest that dual trigger might increase both oocyte and embryo yields, as well as clinical pregnancy rates and live birth rates in patients fulfilling the POSEIDON group 4 criteria. However, large-scale randomized controlled studies are needed to confirm these findings.

## Supporting information

S1 Dataset (XLS)
